# Suicide risk among refugees compared with non-refugee migrants and the Swedish-born majority population

**DOI:** 10.1192/bjp.2019.220

**Published:** 2020-12

**Authors:** Anna-Clara Hollander, Alexandra Pitman, Hugo Sjöqvist, Glyn Lewis, Cecilia Magnusson, James B Kirkbride, Christina Dalman

**Affiliations:** 1 Research Coordinator, Department of Public Health Sciences, Karolinska Institutet, Sweden; 2 Associate Professor in Psychiatry, Division of Psychiatry, University College London, UK; 3 Statistician, Department of Public Health Sciences, Karolinska Institutet, Sweden; 4 Professor, Division of Psychiatry, University College London, UK; 5 Professor, Department of Public Health Sciences, Karolinska Institutet; and Centre for Epidemiology and Community Medicine, Stockholm County Council, Sweden; 6 Reader in Epidemiology, Division of Psychiatry, University College London, UK; 7 Professor, Department of Public Health Sciences, Karolinska Institutet; and Centre for Epidemiology and Community Medicine, Stockholm County Council, Sweden

**Keywords:** Suicide, epidemiology, social deprivation, transcultural psychiatry, mortality

## Abstract

**Background:**

It has been hypothesised that refugees have an increased risk of suicide.

**Aims:**

To investigate whether risk of suicide is higher among refugees compared with non-refugee migrants from the same areas of origin and with the Swedish-born population, and to examine whether suicide rates among migrants converge to the Swedish-born population over time.

**Method:**

A population-based cohort design using linked national registers to follow 1 457 898 people born between 1 January 1970 and 31 December 1984, classified by migrant status as refugees, non-refugee migrants or Swedish-born. Participants were followed from their 16th birthday or date of arrival in Sweden until death, emigration or 31 December 2015, whichever came first. Cox regression models estimated adjusted hazard ratios for suicide by migrant status, controlling for age, gender, region of origin and income.

**Results:**

There were no significant differences in suicide risk between refugee and non-refugee migrants (hazard ratio 1.28, 95% CI 0.93–1.76) and both groups had a lower risk of suicide than Swedish born. During their first 5 years in Sweden no migrants died by suicide; however, after 21–31 years their suicide risk was equivalent to the Swedish-born population (hazard ratio 0.94, 95% CI 0.79–1.22). After adjustment for income this risk was significantly lower for migrants than the Swedish-born population.

**Conclusions:**

Being a refugee was not an additional risk factor for suicide. Our findings regarding temporal changes in suicide risk suggest that acculturation and socioeconomic deprivation may account for a convergence of suicide risk between migrants and the host population over time.

## Background

Internationally, suicide is the 15th most common cause of mortality, accounting for 1.4% of all deaths, and the second leading cause among 15- to 29-year-olds globally.^1^ Risk factors for suicide include low income and unemployment,^2,3^ inherited traits for suicidality,^4^ negative life events^5^ and psychiatric disorders.^6^ Suicide epidemiology is characterised by marked regional variation.^1^ Age-standardised suicide rates are slightly lower in low- and middle-income countries (LMICs) than in high-income countries (11.2 *v.* 12.7 per 100 000 population).^1^ Although data from some LMICs are of poorer quality, and suicide is likely to have been underreported in some of these countries, it is estimated that 75.5% of all suicides globally occur in LMICs because of their relatively larger population size.^1^ Specific suicide risk factors, namely psychiatric disorders and socioeconomic adversity, are more common among migrants in Europe compared with the majority population.^7,8^ Perhaps surprisingly, a systematic review of 24 studies describing suicide epidemiology among migrants to Europe found no consistent pattern of risk.^9^ The study concluded that there is major heterogeneity in suicide risks among immigrants relative to the host populations of European countries, which may depend on a variety of factors. For example, the authors suggested that migrants may ‘import’ high or low levels of suicide risk from their country of origin.^9^ In Sweden and Denmark, first-generation migrants have been shown to have lower suicide risk than the native population,^10,11^ although whether this risk is heterogeneous by factors such as refugee status or time since immigration remains unclear. Refugees, for example, represent a particularly vulnerable group who may have been exposed to additional risk factors for suicide, including traumatic life events^12^ and psychiatric disorders.^13,14^ Because of this, it has been hypothesised that refugees would also exhibit elevated suicide risk compared with non-refugee migrants.^15^ However, because immigrant status is not always subclassified by refugee status,^9^ it has been difficult to examine suicide rates in this group empirically. Higher suicide risk among refugees compared with non-refugee migrants would be consistent with a role for severe past trauma and mental illness in the aetiology of suicide. However, if refugees and non-refugee migrants from the same areas of origin share similar suicide risk, this would strengthen the alternative theory that suicide is strongly influenced by cultural or behavioural factors shared by people of similar backgrounds. This latter theory would also be strengthened if migrant suicide rates were found to converge toward rates for the native population over time, following immigration.

Sweden has a high standard of official population registers adapted for psychiatric research, including data on age, gender, socioeconomic factors and migration-related variables such as refugee status. Since the 1970s Sweden has experienced substantial flows of refugee migrants, and until November 2015, granted more refugee applications *per capita* than any other high-income country. We sought to investigate, in a cohort of 1.46 million people, whether the risk of death by suicide was higher among refugees compared with non-refugee migrants from the same areas of origin and the Swedish-born population, and whether rates among migrants converged to Swedish rates over time. We hypothesised that refugees would have a higher risk of suicide than non-refugee migrants, but lower risks than the native-born Swedish population. We also hypothesised (the convergence hypothesis) that increased time in Sweden (i.e. years from date of immigration) would increase risk of death by suicide among all migrants (both refugees and non-refugee migrants). We also wanted to test whether the hypothesised differences between refugees and non-refugees were spurred by the specific region of origin of different migrant groups.

## Method

### Setting

Data were extracted from a large, longitudinal database of linked national registers, known as Psychiatry Sweden. This includes data on all people officially resident in Sweden after 1 January 1932, linked via a unique personal identity number and anonymised by Statistics Sweden for research purposes. People without an official residence permit in Sweden (i.e. asylum-seekers or undocumented migrants) are not documented in the official registers and were therefore excluded.

### Data sources

Exposure and confounder data were obtained from the Total Population Register, the Multi-generation Register and the immigration and emigration database, which records the reason why a residence permit was granted according to the Swedish Migration Agency's definition of refugee status. In Sweden, people granted asylum as refugees are defined according to Swedish law, based on the United Nations definition of someone who ‘owing to a well-founded fear of being persecuted […] is outside the country of his nationality, and is unable to, or owing to such fear, is unwilling to avail himself of the protection of that country’.^16^ Our outcome, death by suicide, was recorded in the Causes of Death Register.

### Study population

We established a retrospective cohort of all people born between 1 January 1975 and 31 December 1984, which included 2 071 673 individuals. Because refugee status was not available in the Swedish national registers before 1 January 1985, migrants who immigrated to Sweden before this date were excluded (*n* = 68 047, 3.28%). We also excluded adoptees (*n* = 12 875, 0.62%) and children of migrants (persons with one or two parents born abroad; *n* = 266 349, 12.86%). We only included migrants from refugee-generating countries (see supplementary Table 1 available at https://doi.org/10.1192/bjp.2019.220), defined as countries with more than 150 refugee participants identified in our cohort during follow-up. We excluded migrants born in non-refugee-generating countries (*n* = 247 960, 11.97%). We removed those who died before the study period (1991–2015) began (*n* = 12 401, 0.60%) and those who had no reported family income (including wages, welfare benefits, other social subsidies and pensions) during their full study period or had a time-at-risk of <1 year (*n* = 6143, 0.30%). The final cohort size was 1 457 898 participants, followed from their 16th birthday or date of arrival in Sweden, whichever was later, until death by any cause, emigration from Sweden or 31 December 2015, whichever came first.

### Patient involvement

No patients were involved in setting the research question or the outcome measures, nor were they involved in developing plans for design or implementation of the study. No patients were asked to advise on interpretation or writing up of results.

### Outcome

Our outcome was death by suicide defined using ICD-10^17^ codes X60-84 (for suicide) and Y10-34 (for deaths by undetermined intent). Deaths in the registry were included irrespective of whether they had occurred in Sweden or abroad (if a person had left Sweden temporarily and died during this time). Deaths abroad are registered on the Causes of Death Register either with the help of Swedish embassies/consulates, using Swedish death certificates or domestic death certificates. International regulations for death certificates oblige physicians to certify deaths according to World Health Organization standards.

### Exposures

The main exposure was migrant status, with three categories: born in Sweden to two Swedish-born parents (henceforth referred to as Swedish-born), refugees and non-refugee migrants, defined as all other migrants from refugee-generating countries granted official residency in Sweden.

Our second exposure was time in Sweden, defined (in years) as time from date of exit from the cohort minus date of immigration, as recorded in register data. To test convergence, we classified migrants by grouping of years spent in Sweden: 0–5, 6–10, 11–15, 16–20 and 21–31 years post-migration.

### Confounders

We included attained age and gender as *a priori* confounders in all analyses. We used region of origin, defined by country of birth as both a stratification variable (effect modifier) and as a possible confounder. Although Statistics Sweden records data on specific country of birth, information is only released for research purposes according to larger geographical regions to ensure confidentiality (see supplementary Table 1). We used these broad regions of origin in our analysis: sub-Saharan Africa, Asia, Eastern Europe and Russia, and the Middle East and North Africa.

To adjust for possible socioeconomic differences, we included individual disposable family income in Sweden, derived from the longitudinal integration database for health insurance and labour market studies: the LISA database. Disposable income, estimated by Statistics Sweden, was defined as annual disposable income based on total family income from all registered sources, including wages, welfare benefits, other social subsidies and pensions. Individual disposable family income is derived by weighting total family income according to household size and composition, with younger children given lower weights than older household members. We defined income as the individual disposable family income categorised into quintiles, based on the total population for the relevant year. The income variable was used as a time-varying confounder, varying for each year, and used with a 1-year lag to account for suicide events early in the year.

### Ethics statement

The authors assert that all procedures contributing to this work comply with the ethical standards of the relevant national and institutional committees on human experimentation and with the Helsinki Declaration of 1975, as revised in 2008. This research has ethical approval as part of Psychiatry Sweden ‘Psykisk ohälsa, psykiatrisk sjukdom: förekomst och etiologi’ approved by the Stockholm Regional Ethical Review Board (number 2010/1185-31/5).

### Statistical analysis

We reported basic descriptive statistics for refugees, non-refugee migrants and the Swedish-born native population (Table 1). We fitted time-dependent Cox models to estimate hazard ratios for suicide and 95% confidence intervals according to our main exposure variables. Follow-up time used age as an underlying time-scale, whereby the primary time variable in the Cox model is defined by participants’ age (in years) at entry into the follow-up period, and the age at which they experienced an event or their follow-up was censored. Use of the age scale controls for age effects and provides a meaningful basis on which to examine how risk varies over increasing age.

**Table 1:** Cohort characteristics by migrant status: refugees, non-refugee migrants and Swedish-born population

		Swedish-born	Non-refugee migrants	Refugees	Total
Total	*N* (%)	1 224 948 (84.02)	194 827 (13.36)	38 123 (2.61)	1 457 898 (100)
	Cases (%)	3536 (0.29)	160 (0.08)	51 (0.13)	3747 (0.26)
Age					
At entry	Mean (s.d.)	16.93 (1.51)	25.13 (6.00)	24.75 (6.96)	18.23 (4.11)
At exit	Mean (s.d.)	38.46 (4.76)	36.67 (5.22)	37.76 (4.79)	38.20 (4.86)
Gender					
Men	*n* (%)	629 435 (84.89)	89 536 (12.08)	22 487 (3.03)	741 458 (100)
	Cases (%)	2600 (0.41)	105 (0.12)	43 (0.19)	2748 (0.37)
Women	*n* (%)	595 513 (83.12)	105 291 (14.70)	15 636 (2.18)	716 440 (100)
	Cases (%)	936 (0.16)	55 (0.05)	8 (0.05)	999 (0.14)
Area of origin					
Swedish-born	*n* (%)	1 224 948 (100)			1 224 948 (100)
	Cases (%)	3536 (0.29)			3536 (0.29)
Sub-Saharan Africa	*n* (%)		17 736 (66.50)	8935 (33.50)	26 671 (100)
	Cases (%)		21 (0.12)	8 (0.09)	29 (0.11)
Asia	*n* (%)		67 193 (94.03)	4265 (5.97)	71 458 (100)
	Cases (%)		37 (0.06)	6 (0.14)	43 (0.06)
Eastern Europe and Russia	*n* (%)		56 579 (93.07)	4213 (6.93)	60 792 (100)
	Cases (%)		56 (0.10)	6 (0.14)	62 (0.10)
The Middle East and North Africa	*n* (%)		53 319 (72.02)	20 710 (27.98)	74 029 (100)
	Cases (%)		46 (0.09)	31 (0.15)	77 (0.1)
Disposable income (in quintiles)					
Highest quintile	*n* (%)	28 457 (93.69)	1898 (6.25)	22 (0.07)	30 372 (100)
	Cases (%)	66 (0.23)	0 (0)	0 (0)	66 (0.22)
Higher medium quintile	*n* (%)	299 472 (97.06)	8312 (2.69)	829 (0.27)	308 552 (100)
	Cases (%)	416 (0.14)	5 (0.06)	2 (0.24)	423 (0.14)
Medium quintile	*n* (%)	526 689 (94.09)	28 449 (5.08)	4763 (0.85)	559 784 (100)
	Cases (%)	993 (0.19)	6 (0.02)	4 (0.08)	1003 (0.18)
Lower medium quintile	*n* (%)	312 973 (82.21)	56 643 (14.88)	11 217 (2.95)	380 718 (100)
	Cases (%)	1341 (0.43)	63 (0.11)	17 (0.15)	1421 (0.37)
Lowest quintile	*n* (%)	57 357 (32.21)	99 525 (55.88)	21 292 (11.96)	178 091 (100)
	Cases (%)	720 (1.26)	86 (0.09)	28 (0.13)	834 (0.47)
Suicide/death by undetermined intent					
Total *n* cases	Cases (%)	3536 (100)	160 (100)	51 (100)	3747 (100)
X60-84 (suicide)	Cases (%)	2674 (75.62)	127 (79.38)	35 (68.63)	2836 (75.69)
Y10-34 (deaths by undetermined intent)	Cases (%)	862 (24.38)	33 (20.63)	16 (31.37)	911 (24.31)

Values are shown as numbers (percentages), unless otherwise indicated.

In our first analysis, we estimated risk of death by suicide in refugees relative to non-refugee migrants adjusted for gender as well as disposable income. In our second analysis, we estimated risk of death by suicide in refugees and non-refugee migrants relative to the Swedish-born group, adjusted for gender and disposable income. Next, we determined whether risk of death by suicide in refugees relative to non-refugee migrants differed by region of origin, by stratifying by region of origin. We then tested for differences in suicide risk by region of origin in the total population, including refugees, non-refugees and Swedish-born residents. Finally, we tested whether risk of suicide among migrants (both refugees and non-refugees) converged to the level of risk in the Swedish-born population based on time since immigration. We also tested whether the possible convergence differed for refugees and non-refugees and men and women, using nested likelihood ratio test. For all the above models, we tested, using a nested likelihood ratio test, whether the relation between exposure and outcome differed between men and women by fitting an interaction term between exposure and gender, presenting stratum-specific findings to investigate whether risk estimates were modified.

We performed three sensitivity analyses. The first sensitivity analysis explored whether mortality in the total population might have been underestimated because of the possibility that people had left Sweden temporarily, without being recorded as having emigrated, and died abroad, thus unrecorded by Statistics Sweden, and whether this possible underestimation might be differential. To test for this potential bias, we used a method developed by Weitoft *et al*^18^ using fiscal or welfare transactions with the State as an indicator of residence in Sweden. If a person did not have any benefits (e.g. universal child allowance or disability pension) or tax transactions with the State for three consecutive years, this person would be likely to have left the country without informing the tax authorities. We tested the effect of excluding this group, which would include those with unrecorded deaths outside Sweden, to compare this finding with our main findings. We also ran the analysis on the excluded group only.

A second sensitivity analysis tested whether differences in risks of dying by causes other than suicide (competing risks theory) might explain potential differences in suicide risk between the groups; that is, if refugees were at higher risk of dying by non-suicide causes than by suicide, this could mask a significantly higher risk of dying by suicide in refugees than in the other groups. We calculated if competing risks of mortality due to other causes than suicide could explain potential differences in risk of death by suicide.

The third sensitivity analysis tested if the results could be attributable to outcome misclassification. In the main analysis we defined death by suicide using both ICD-10 codes X60-84 (for suicide) and Y10-34 (for deaths by undetermined intent). However, as there might be differences in classification of the outcome by migrant status, we estimated risk of death by confirmed suicides (X60-84) and for deaths by undetermined intent (Y10-34) separately in refugees and non-refugee migrants relative to the Swedish-born group, adjusted for gender and disposable income.

Finally, we tested the assumption of proportional hazards in our main Cox regression models, using graphical and Schoenfeld residuals tests, for the fixed confounders and exposures. All analyses were conducted with the SAS software package version 9.4 for Windows.

### Data availability

The statistical code is available from the corresponding author. Under Swedish law and ethical approval, patient-level data cannot be made available.

## Results

Of almost 1.46 million study participants aged 16–43 years, 3747 died by suicide during the follow-up period (cumulative incidence 0.22%, 95% CI 0.21–0.23). Median age for death by suicide was 30 years for Swedish-born residents (5th–95th percentile 29.08–30.92), 32 years for non-refugee migrants (5th–95th percentile 31.35–32.65) and 32 years for refugees (5th to 95th percentile 30.77–33.23). Mean time to death from the start of the study was 13.48 years for Swedish-born individuals (s.d. 6.95), 10.85 years for refugees (s.d. 6.48) and 10.09 years for non-refugee migrants (s.d. 6.01), with significant differences found using the Kruskal–Wallis test (*P* < 0.001). When comparing the refugees with the non-refugees only the test did not identify significant differences between the two groups (*P* = 0.164). We found significant differences in the proportions of Swedish-born residents, refugees and non-refugee migrants who had died by suicide (X60-84), as compared with deaths by undetermined intent (Y10-34) (*P* < 0.001), with a larger share of deaths by undetermined intent among refugees (see Table 1). For other characteristics of the study population, see Table 1. For the numbers of refugees and non-refugee migrants from specific refugee-generating countries see supplementary Table 1.

We found no evidence to support any major differences in risk of suicide between refugee and non-refugee migrants after adjustment for age and gender (hazard ratio 1.28, 95% CI 0.93–1.76) (see Table 2, model 1) or with additional adjustments for disposable income (hazard ratio 1.28, 95% CI 0.93–1.76) (see Table 2, model 2). There was no evidence of statistical interaction between gender and migrant status on suicide risk (*P* > 0.1). Initial results suggested that refugees and non-refugee migrants were at lower risk of suicide than the Swedish-born, after adjustment for age and gender (refugees: hazard ratio 0.70, 95% CI 0.53–0.92; non-refugees: hazard ratio 0.55, 95% CI 0.47–0.64) (see Table 3, model 1); this pattern persisted after additional adjustment for disposable income (see Table 3, model 2). As before, there was no evidence of statistical interaction between gender and migrant status on suicide risk (*P* > 0.1).

**Table 2:** Hazard ratios and 95% confidence intervals for the risk of death by suicide in the total population for refugees and non-refugee migrants

	Model 1^a^	Model 2^b^
Migrant status		
Non-refugee migrant (reference)	1	1
Refugees	1.28 (0.93–1.76)	1.28 (0.93–1.76)
Gender		
Men (reference)	1	1
Women	0.39 (0.29–0.52)	0.38 (0.28–0.52)
Disposable income (in quintiles)		
Highest quintile		0.42 (0.17–1.02)
Higher medium quintile		1.03 (0.57–1.89)
Medium quintile (reference)		1
Lower medium quintile		1.17 (0.70–1.96)
Lowest quintile		1.46 (0.94–2.26)

a. Adjusted for attained age and gender.

b. Adjusted for attained age. Gender and total family disposable income adjusted for family size.

**Table 3:** Hazard ratios and 95% confidence intervals for the risk of death by suicide in the total population for Swedish-born, refugees and non-refugee migrants

	Model 1^a^	Model 2^b^
Migrant status		
Swedish-born (reference)	1	1
Non-refugee migrant	0.55 (0.47–0.64)	0.35 (0.30–0.42)
Refugees	0.70 (0.53–0.92)	0.44 (0.33–0.58)
Gender		
Men (reference)	1	1
Women	0.38 (0.36–0.41)	0.35 (0.33–0.38)
Disposable income (in quintiles)		
Highest quintile		0.66 (0.59–0.75)
Higher medium quintile		0.95 (0.85–1.06)
Medium quintile (reference)		1
Lower medium quintile		1.50 (1.35–1.66)
Lowest quintile		2.80 (2.55–3.08)

a. Adjusted for attained age and gender.

b. Adjusted for attained age. Gender and total family disposable income adjusted for family size.

We found no evidence these patterns differed by region of origin in stratified analyses (see supplementary Table 2). As such, we considered refugees and non-refugee migrants as a single group to test whether suicide risk varied by region of origin relative to the Swedish-born population. In these analyses, suicide risk appeared to vary by region of origin and gender (*P* = 0.03). Thus, for men, migrants from all regions of origin except sub-Saharan Africa were at lower suicide risk than Swedish-born. By contrast, only women from Asia were found to have lower risks than Swedish-born residents (see supplementary Table 3, model 1), although men and women from all regions of origin had lower risks than the Swedish-born after additional adjustment for income (supplementary Table 3, model 2).

**Fig. 1:**
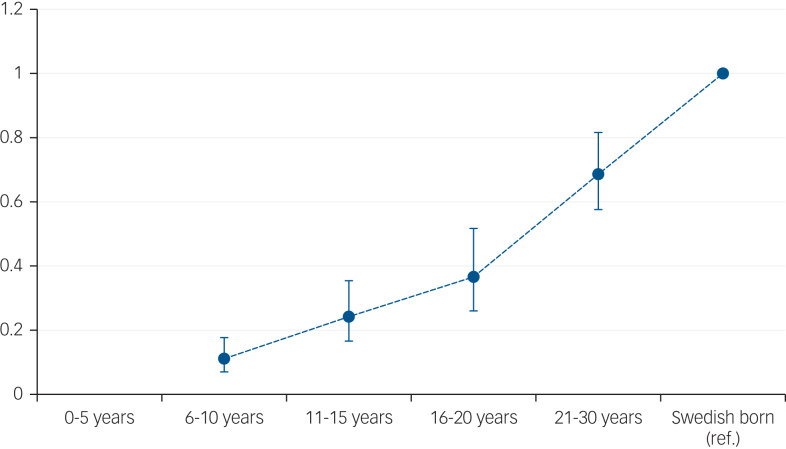
Hazard ratios and 95% confidence intervals for the risk of death by suicide in the total population for migrants, based on years in Sweden compared with Swedish-born natives.

After adjustment for age and gender, there was evidence supporting convergence to the risk of suicide in the Swedish-born group by time since immigration, such that those migrants living in Sweden for the longest time (21–31 years) had similar risks to the Swedish-born (supplementary Table 4, model 1 and Fig. 1). This pattern was also apparent after adjustment for disposable income, although migrants remained at lower suicide risk than the Swedish-born population at all times from immigration, (supplementary Table 4, model 2). These patterns did not differ between refugees and non-refugee migrants (*P* > 0.1) or between men and women (*P* > 0.1).

### Sensitivity analyses

The first sensitivity analysis tested whether individuals who may have left Sweden before censorship (*n* = 23 576, 1.617%) without informing authorities had biased our findings. When excluding this group our estimates were unchanged. We also performed the analysis on the excluded group (see supplementary Table 5). The second sensitivity test examined whether competing risk of death from causes other than suicide might have affected our results. Refugees and non-refugee migrants had similar risk of death owing to causes other than suicide (see supplementary Tables 6 and 7). The third sensitivity analysis estimated risk of death by confirmed suicides (X60-84) and for deaths by undetermined intent (Y10-34) separately in refugees and non-refugee migrants relative to the Swedish-born group, adjusted for gender and disposable income. When using confirmed suicides only, the results were unchanged, see supplementary Table 8.

The assumption of proportional hazards in the Cox model was not violated.

## Discussion

In the first study to disentangle risk of death by suicide among refugees compared with non-refugee migrants, we found little evidence to support the often-stated hypothesis^15^ of increased risk of suicide in refugees. After adjustment for attained age, gender and disposable income, risk of death by suicide was reduced by similar amounts in refugees (hazard ratio 0.44, 95% CI 0.33–0.58) and non-refugee migrants (hazard ratio 0.35, 95% CI 0.30–0.42) compared with the Swedish-born population. However, we found strong evidence that rates in migrants increased over time and finally converged to the rate for Swedish-born residents.

### Strengths and weaknesses

This study has several methodological strengths. It was based on a large, national total population cohort of almost 1.5 million people, with up to 31 years of follow-up, using linked Swedish register data extracted from Psychiatry Sweden. Psychiatry Sweden has previously formed the basis for cohort studies describing differences in non-affective psychotic disorders between refugees and other migrants.^14^ Swedish register data are known to be reliable for research purposes,^19^ including classifying suicide deaths.^20^ A study of this kind has not previously been possible owing to a lack of power (suicide is relatively rare in many samples) and information describing reason for migration in Swedish, and other, population-based data-sets. Our exposure variable of migration status, qualified as refugee or non-refugee, was defined according to the Swedish Migration Agency classifications, with the advantage that they are based on reasons for a person being granted a residence permit in Sweden according to Swedish rules and regulations. During the years covered by this study, Sweden was relatively restrictive in granting asylum, with the threshold of proof for gaining asylum requiring the applicant to provide adequate evidence. Thus, the sensitivity of the refugee status classification is likely to be high. It is possible that the non-refugee category could include misclassified refugees, reducing its specificity and weakening the association between migration status and suicide. However, we have previously found this classification to be a valid means of distinguishing between the two groups,^14,15,20^ hence we would not expect misclassification bias to have affected the results to any major extent. Sensitivity analyses suggested that our results were not attributable to selective emigration or competing mortality risks. Risk of suicide increased with age in Sweden. Despite studying this comprehensive cohort with a follow-up of 31 years, the lack of evidence of a difference (with wide confidence intervals) could still be attributable to limited follow-up time or limited numbers. Lack of power is unlikely as the cohort included the total population of refugees and non-refugee migrants from refugee generating countries and not a drawn sample of refugees. Date of residence permit is not the same as date of physical entry to Sweden and, for some refugees, there is a lag between entering Sweden to obtaining a residence permit. However, as there were no suicides among migrants during their first years in Sweden, this should have a small overall effect. We found differences in the proportions of Swedish-born residents, refugees and non-refugee migrants who had died by suicide (X60-84), as compared with those whose deaths were classified as by undetermined intent (Y10-34), with a significantly larger share of undetermined deaths among refugees (*P* < 0.001). Sensitivity analysis showed that using confirmed suicides only gives the same results as using both outcomes combined.

### The findings in relation to other studies

Our findings suggest that despite refugees having a high prevalence of psychiatric disorders^7^ and greater exposure to past traumatic life events,^12^ both being risk factors for suicide, refugees in our cohort covering 31 years of follow-up do not have a higher risk of suicide than non-refugee migrants, even from the same region of origin. This seems at odds with the literature regarding psychiatric disorders and traumatic life events as risk factors of suicide. One explanation is that studies of negative life events have tended to focus on personal stressors, such as interpersonal conflicts with family members or friends,^5^ that may not be randomly distributed (e.g. accumulate in vulnerable families). Studies measuring the effect of collectively experienced life events such as war or natural disasters, are more problematic methodologically and findings often conflict.^21,22^ This may be because collective stressors are less harmful as a risk factor for suicide than interpersonal ones, or because both vulnerable and less vulnerable individuals are exposed to collective stressors, which are more randomly assigned. Although refugees have an increased risk of having experienced traumas,^12^ we did not measure trauma specifically in this study, hence we have only tested the ecological assumption that refugees have often been exposed to traumas. One reason that the known risk factors for suicide in the general population did not seem to create an increased risk among refugees may be resilience in the refugee group. Being able to identify strong reasons for living (encompassing survival and coping beliefs) has been found to be protective against suicidal thoughts and behaviours, and is likely to be linked to resilience factors.^23^ Persons who decide to flee persecution (i.e. refugees) could possibly be a group with particularly strong survival and coping beliefs.

Our study strengthens the theory that suicide is strongly influenced by cultural factors that diminish over time (convergence) and social adversity, because adjusting for disposable income attenuated the convergence. In terms of suicide risk by region of origin, no group had a higher risk than the Swedish-born population. After adjustment for disposable income migrants groups of all regions had lower suicide rates than the Swedish-born. These findings are in line with previous findings that migrants have a lower suicide rate than the native population^10,11^ and that accumulated time in a host country is a risk factor for suicide rate converging to the level of the native population among migrants.^24,25^ The lower rates in migrants have been suggested to be due to cultural differences in attitudes toward suicide (i.e. that migrants ‘import’ their suicide risk from their country of origin), and this is supported by our results.^9^ The observed convergence was reduced when adjusted for disposable income, and this is also in line with previous studies in which social adversity has been shown to be a risk factor for suicide.^2,3^ All refugees in this study had residence permits, thus excluding asylum-seekers. Hence our findings cannot be generalised to asylum-seekers. A study from 2019 showed that the suicide rate among asylum-seeking unaccompanied refugee minors was almost tenfold that for Swedish youth.^26^ The differences between the high suicide rates among asylum seekers in the report^26^ and the present study might be because the refugees included in this study had been granted residence permit and hence were in a secure position in life, pointing again to the balance of risk and protective factors.

### Meaning of the study: possible explanations and implications for clinicians and policymakers

The World Health Organization highlights the specific difficulties faced by refugees, asylum-seekers and migrants, including stigma, and the stresses of acculturation and dislocation.^27^ Although refugee status is associated with traumatic experiences and psychiatric disorders, our study suggests that as opposed to asylum seekers, refugees in Sweden do not have an elevated risk of suicide compared with non-refugee migrants from the same area of origin, and that both groups have a lower risk of suicide than the Swedish-born population. There are cultural aspects of suicide risk for migrants and the risks converge over time, suggesting that acculturation and social deprivation possibly influence suicide risk more than refugee status. The balance between risk and protective factors is crucial for the risk of suicide. Migrant history could be an important aspect of history-taking in clinical settings, accompanied by an awareness that migration history may influence suicide risk for more than two decades in a country.

### Unanswered questions and future research

This study has raised several new questions on the role of trauma and mental illness as risk factors for suicide in general, and the role of acculturation and social deprivation as risk factors for suicide among migrants. Future studies need to explore these factors, assisting efforts to delineate risk factors of suicide in both migrants and the general population.

## Supplementary material

For supplementary material accompanying this paper visit https://doi.org/10.1192/bjp.2019.220. click here to view supplementary material
