# Supplementary material for: Suicide risk among refugees compared with non-refugee migrants and the Swedish-born majority population
Source: Br J Psychiatry. 2019 Oct 14;217(6):686–92. doi: 10.1192/bjp.2019.220 (PMC7705666; doi:10.1192/bjp.2019.220)
Supplement: Supplementary file 1 [file S0007125019002204sup001.docx]

Supplementary table 1: Relationship between country of birth and region

| Country | Sub-Saharan Africa | Asia | Eastern Europe and Russia | The Middle East |
| --- | --- | --- | --- | --- |
| North Asia |  | 15,944 (6.84 %) |  |  |
| Southeast Asia |  | 25,269 (10.85 %) |  |  |
| Central Asia |  | 30,245 (12.98 %) |  |  |
| Baltics and Russia |  |  | 16,954 (7.28 %) |  |
| Former Yugoslavia |  |  | 43,838 (18.82 %) |  |
| Iraq |  |  |  | 42,226 (18.13 %) |
| Iran |  |  |  | 23,084 (9.91 %) |
| North Africa |  |  |  | 8,719 (3.74 %) |
| Somalia, Eritrera, Etiopien, Djibouti | 26,671 (11.45 %) |  |  |  |
| Total 232,950 (100.00 %) | 26,671 (11.45 %) | 71,458 (30.68 %) | 60,792 (26.10 %) | 74,029 (31.78 %) |

| Supplementary table 2: Hazard Ratios (HR) and Confidence Interval (CI) for risk of death by suicide in the migrant sub-group, stratified by country of origin, for refugees and non-refugees. | |
| --- | --- |
| **Region of origin** | **HR (CI)* for refugees**** |
| Sub-Saharan Africa | 1.00 (0.44-2.28) |
| Asia | 1.39 (0.58-3.35) |
| Eastern Europe and Russia | 1.14 (0.49-2.65) |
| The Middle East | 1.22 (0.77-1.93) |
| * Adjusted for age as an underlying time-scale and sex ** The reference is non-refugee immigrants. | |

Supplementary table 3: Hazard Ratios (HR) and Confidence Intervals (CI) for risk of death by suicide in the cohort by region of origin for men and women

|  | **Men** |  | **Women** |  |
| --- | --- | --- | --- | --- |
|  | Model 1* | Model 2** | Model 1* | Model 2** |
| **Region of origin** |  |  |  |  |
| Swedish-born (reference) | 1 | 1 | 1 | 1 |
| Sub-Saharan Africa | 0.83 (0.55-1.24) | 0.47 (0.31-0.70) | 0.41 (0.17-0.98) | 0.25 (0.10-0.60) |
| Asia | 0.42 (0.27-0.63) | 0.24 (0.16-0.36) | 0.69 (0.44-1.06) | 0.49 (0.31-0.75) |
| Eastern Europe and Russia | 0.50 (0.37-0.69) | 0.35 (0.25-0.48) | 0.69 (0.45-1.04) | 0.53 (0.35-0.80) |
| The Middle East | 0.67 (0.52-0.85) | 0.41 (0.32-0.52) | 0.41 (0.24-0.70) | 0.28 (0.16-0.47) |
| **Disposable income (in quintiles)** |  |  |  |  |
| Highest quintile |  | 0.66 (0.58-0.76) |  | 0.66 (0.50-0.87) |
| Higher medium quintile |  | 0.96 (0.84-1.09) |  | 0.91 (0.73-1.13) |
| Medium quintile (reference) |  | 1 |  | 1 |
| Lower medium quintile |  | 1.4 (1.24-1.58) |  | 1.75 (1.45-2.11) |
| Lowest quintile |  | 2.90 (2.6-3.25) |  | 2.56 (2.14-3.08) |

* adjusted for attained age.

**adjusted for attained age and disposable income.

Supplementary Table 4: Hazard Ratios (HR) and 95 % Confidence Interval (CI) for the risk of death by suicide in the total population for migrants, based on years in Sweden compared with Swedish-born natives

| **HR (CI)** | **Model 1*** | **Model 2**** |
| --- | --- | --- |
| **Time in Sweden** |  |  |
| Swedish-born (reference) | 1 | 1 |
| 0-5 years in Sweden | n.a.*** | n.a. |
| 6-10 years in Sweden | 0.20 (0.13-0.32) | 0.11 (0.07-0.18) |
| 11-15 years in Sweden | 0.40 (0.27-0.58) | 0.24 (0.17-0.35) |
| 16-20 years in Sweden | 0.58 (0.41-0.81) | 0.37 (0.26-0.52) |
| 21-31 years in Sweden | 0.94 (0.79-1.12) | 0.69 (0.58-0.82) |
| **Sex** |  |  |
| Men (reference) | 1 | 1 |
| Women | 0.38 (0.36-0.41) | 0.35 (0.33-0.38) |
| **Disposable income (in quintiles)** |  |  |
| Highest quintile |  | 0.66 (0.59-0.75) |
| Higher medium quintile |  | 0.95 (0.85-1.06) |
| Medium quintile (reference) |  | 1 |
| Lower medium quintile |  | 1.50 (1.35-1.66) |
| Lowest quintile |  | 2.84 (2.59-3.13) |
| * adjusted for attained age and sex ** adjusted for attained age, sex and total family disposable income adjusted for family size *** not available as no migrants in our sample died by suicide during the 0-5 years period. | | |

Supplementary Table 5: Hazard Ratios (HR) and Confidence Interval (CI) for the risk of death by suicide in the total population for Swedish born, refugees and non-refugees. The sample is those who had a missing reported income during their study period for 3 years in row.

| **HR (CI)** | **Model 1*** | **Model 2**** |
| --- | --- | --- |
| **Migrant Status** |  |  |
| Swedish-born (reference) | 1 | 1 |
| Non-refugee migrant | 2.00 (0.44-9.12) | 1.57 (0.32-7.64) |
| Refugees | N/A | N/A |
| **Sex** |  |  |
| Men (reference) | 1 | 1 |
| Women | 0.27 (0.07-1.00) | 0.23 (0.06-0.86) |
| **Disposable income (in quintiles)** |  |  |
| Highest quintile |  | N/A |
| Higher medium quintile |  | 0.18 (0.02-1.53) |
| Medium quintile (reference) |  | 1 |
| Lower medium quintile |  | 0.49 (0.09-2.52) |
| Lowest quintile |  | 0.54 (0.14-2.10) |
| * adjusted for attained age and sex ** adjusted for attained age. sex and total family disposable income adjusted for family size | | |

Supplementary table 6: Suicide and other causes of death against Swedish-born, immigrant and refugees (row percentage)

|  | **Swedish-born** | **Migrant** | **Refugee** | **Total** |
| --- | --- | --- | --- | --- |
| **Suicide** | 9,821 (91.9 %) | 702 (6.57 %) | 167 (1.56 %) | 10,690 (72.9 %)* |
| **Other causes** | 3,747 (94.3 %) | 169 (4.25 %) | 56 (1.41 %) | 3,972 (27.1 %)* |
| **Total** | 13,568 (92.54 %) | 871 (5.94 %) | 223 (1.52 %) | 14,662 (100%) |
| * Column percentage | | | | |

Supplementary table 7: Hazard Ratios (HR) and Confidence Interval (CI) for the risk of death due to other causes

|  | Model 1* | Model 2** |
| --- | --- | --- |
| **Death by other causes than suicide** |  |  |
| Swedish-born | 1 | 1 |
| Refugees | 0.80 (0.69-0.93) | 0.56 (0.48-0.65) |
| Non-refugees | 0.82 (0.76-0.89) | 0.59 (0.55-0.64) |

*Model 1: crude HR for all, adjusted for attained age, sex.

**Model 2: HRs for all, adjusted for attained age, sex, and disposable income.

Supplementary table 8: Hazard Ratios (HR) and Confidence Interval (CI) for the risk of death by confirmed suicide X60-84 and the risk of deaths by undetermined intent Y10-34 separately, adjusted for attained age, sex, and disposable income

| **Death by confirmed suicide X60-84** |  |
| --- | --- |
| Swedish-born | 1 |
| Refugees | 0.38 (0.31-0.46) |
| Non-refugees | 0.41 (0.30-0.58) |
| **Deaths by undetermined intent Y10-34** |  |
| Swedish-born | 1 |
| Refugees | 0.26 (0.18-0.37) |
| Non-refugees | 0.47 (0.28-0.77) |
